# Supplementary material for: On the renormalization group fixed point of the two-dimensional Ising model at criticality
Source: Sci Rep. 2023 Sep 8;13:14859. doi: 10.1038/s41598-023-42005-4 (PMC10491843; doi:10.1038/s41598-023-42005-4)
Supplement: Supplementary file 1 — Supplementary Information. [file 41598_2023_42005_MOESM1_ESM.pdf]

Supplementary material for "On the renormalization group fixed point of the two-dimensional Ising model at criticality"

Alexander Stottmeister[1] and Tobias J. Osborne[1]

[1] *Institut für Theoretische Physik, Leibniz Universität Hannover, Appelstr. 2, 30167 Hannover, Germany*

COARSE GRAINING $\mathcal{E}$: SPIN CHAIN REPRESENTATION

Let us briefly explain the structure of the coarse graining channel $\mathcal{E}$ in the spin chain representation (additional details can be found in [1]): For finite-length spin chains, the Jordan-Wigner transformation,

$$a_j = \Big(\prod_{-M\leq l<j} \sigma_l^{(1)}\Big)\tfrac{1}{2}(\sigma_j^{(3)} + i\sigma_j^{(2)}), \qquad j \in \mathbb{Z} \tag{1}$$

provides a $*$-isomorphism between the Pauli algebra $\mathcal{P}_M$ and the complex fermion algebra $\mathfrak{A}_M$. In terms of Araki's self-dual field [2], $\Psi(\xi,\eta) = a(\xi - i\eta) + a^\dagger(\overline{\xi + i\eta})$, the inverse of this transformation can be written as:

$$\sigma_j^{(3)} = \Big(\prod_{-M\leq l<j} \Psi(\delta_l, 0)\Psi(0, i\delta_l)\Big)\Psi(\delta_j, 0), \qquad \sigma_j^{(1)} = \Psi(\delta_j, 0)\Psi(0, i\delta_j). \tag{2}$$

We can now exploit the Jordan-Wigner transformation to find an explicit representation of the dual renormalization group channel α given in Eq. (6) of the main document.First, we observe the associated state correspondence between the natural representations of $\mathcal{P}_M$ on $\mathcal{H}_M = \otimes_{j=-M}^{M-1}\mathbb{C}^2$ and $\mathfrak{A}_M$ on the anti-symmetric Fock space $\mathfrak{F}_-(\mathfrak{h}_M)$:

$$\begin{aligned}|j_1,...,j_n\rangle = a_{j_1}^\dagger ... a_{j_n}^\dagger \Omega_M &= (-1)^{|j|}|-\rangle_{-M}\otimes ...\otimes|+\rangle_{j_1}\otimes ...\otimes|+\rangle_{j_n}\otimes ...\otimes|-\rangle_{M-1} \\ &= (-1)^{|j|}|-_{-M},...,+_{j_1},...,+_{j_n},...,-_{M-1}\rangle,\end{aligned} \tag{3}$$

for $-M \leq j_1 < ... < j_n \leq M-1$, where $\Omega_M = \otimes_{j=-M}^{M-1}|-\rangle = |-,...,-\rangle$ is the Fock vacuum, $|j| = (-1)^{\sum_{i=1}^n j_i}$, and $|\pm\rangle$ are the ± 1-eigenstates of $\sigma^{(1)}$. Second, we decompose the one-particle isometry $R = u_{2M}\circ\iota$ given in Eq. (7) of the main document into a unitary u_{2M} on $\mathfrak{h}_{2M}$ and the trivial embedding $\iota(\mathfrak{h}_M)\subset\mathfrak{h}_{2M}$ given by the identification of the scaled lattice $2\Lambda_M$ as sublattice of Λ_{2M}. Clearly, the unitary is not uniquely fixed on the orthogonal complement of $\iota(\mathfrak{h}_M)$, but there is a natural choice associated with a length-$2N$ low-pass filter $\{h_n\}$ given by a corresponding high-pass filter $\{g_n = (-1)^n\bar{h}_{-n+1+2N}\}$ [3]:

$$(u_{2M}\xi)_j = \sum_{l\in 2\Lambda_M}\xi_l\sum_{n\in\mathbb{Z}}h_n\delta_{l+n,j} + \sum_{l'\in\Lambda_{2M}\setminus 2\Lambda_M}\xi_{l'}\sum_{n\in\mathbb{Z}}g_{n+1}\delta_{l'+n,j}, \qquad \xi\in\mathfrak{h}_{2M}. \tag{4}$$

This choice precisely corresponds to the representation of the discrete wavelet transform (on $L^2(S^1)$ or $L^2(\mathbb{R})$) as a unitary circuit by Evenbly-White [4]. We note that by construction the unitary $U_{2M} = \Gamma(u_{2M})$ in Fig. 3 of the main document acting on $\mathcal{H}_{2M} = \mathfrak{F}_-(\mathfrak{h}_{2M})$ is given

by the multiplicative second quantization of u_M, while the unital $*$-morphism $\Gamma(\iota)$ is dual to the partial trace $\mathrm{ptr}:\mathcal{H}_{2M}\rightarrow\mathcal{H}_M$. In the limit $M\rightarrow\infty$, (4) is still meaningful if the low-pass filter is considered in non-periodic form as arising from a scaling function $s\in\mathbb{C}^r(\mathbb{R})$. Similarly, the partial trace is performed with respect to every other site of the infinite-length spin chain. In summary we have,

$$\mathcal{E}=\mathrm{ptr}\circ\mathrm{Ad}_U, \tag{5}$$

and U has the following matrix elements with respect to the $\sigma^{(1)}$-basis:

$$\langle -\ldots+_{j'_1}\ldots+_{j'_n}\ldots-|U|-\ldots+_{j_1}\ldots+_{j_m}\ldots-\rangle=\delta_{n,m}(-1)^{|j'|+|j|}\sum_{\{n\}}\prod_{l=1}^{n}c_{n_l}^{(j_l)}\delta_{j'_l,j_l+n_l}, \tag{6}$$

where $c_n^{(j)}=\begin{cases} h_n & : \quad j\in 2\mathbb{Z} \\ g_{n+1} & : \ j\in\mathbb{Z}\setminus 2\mathbb{Z}\end{cases}$.

EVEN STATES: SPIN-SPIN CORRELATION FUNCTIONS

We provide additional details on the computation of spin-spin correlation functions and the definition of dynamics on the Pauli algebra $\mathcal{P}$ following [2, 5]. The Pauli algebra $\mathcal{P}$ and the fermion algebra $\mathfrak{A}$ are both $\mathbb{Z}_2$-graded in a compatible way, i.e. we have order-2 automorphisms Θ of $\mathcal{P}$ and $\mathfrak{A}$ (denoted by the same symbol) given by:

$$\begin{aligned} &\Theta(\sigma_j^{(1)})=\sigma_j^{(1)}, && \Theta(\sigma_j^{(3)})=-\sigma_j^{(3)}, \\ &\Theta(a(\xi))=-a(\xi), && \xi\in\mathfrak{h}. \end{aligned} \tag{7}$$

It is known that there is a bijective correspondence between even states, those invariant under Θ, on $\mathcal{P}$ and $\mathfrak{A}$, and it is easy to see that the Gibbs states ω_β as well as the ground state ω_∞ are even states on $\mathfrak{A}$ [6]. While the Jordan-Wigner transformation (1) does not extend to a $*$-isomorphism between the infinite-length Pauli algebra $\mathcal{P}$ and the complex fermion algebra $\mathfrak{A}$, it does so for the even parts $\mathcal{P}^{(0)}$ and $\mathfrak{A}^{(0)}$ with respect to the $\mathbb{Z}_2$-grading Θ. In particular, we have:

$$\sigma_j^{(3)}\sigma_{j'}^{(3)}=(a_j+a_j^\dagger)\Big(\prod_{j\le l<j'}[a_l^\dagger,a_l]\Big)(a_{j'}+a_{j'}^\dagger)=\prod_{j\le l<j'}(a_l-a_l^\dagger)(a_{l+1}+a_{l+1}^\dagger)=\prod_{j\le l<j'}\Psi(0,i\delta_l)\Psi(\delta_{l+1},0), \tag{8}$$

for $j\le j'$, where $\Psi(\xi,\eta)=a(\xi-i\eta)+a^\dagger(\overline{\xi+i\eta})$ is Araki's self-dual field. This allows for a direct evaluation of even quasi-free states, e.g., ω_β and ω, on $\mathcal{P}$ in terms of $\mathfrak{A}$: By means of (8) we

obtain the following expression for n-point spin-spin correlation functions (cp. Eq. (18) in the main document):

$$\omega(\sigma^{(3)}_{j_1}...\sigma^{(3)}_{j_{2n}}) = \omega\Big(\prod_{j_1\leq l<j_2}\Psi(0,i\delta_{l_1})\Psi(\delta_{l_1+1},0)...\prod_{j_{2n-1}\leq l<j_{2n}}\Psi(0,i\delta_{l_n})\Psi(\delta_{l_n+1},0)\Big), \tag{9}$$
$$\omega(\sigma^{(3)}_{j_1}...\sigma^{(3)}_{j_{2n+1}}) = 0,$$

for $j_1 \leq ... \leq j_{2n} \leq j_{2n+1}$, which can be evaluated for quasi-free states on $\mathfrak{A}$ as these are determined by their two-point function [6] (odd correlators vanish identically):

$$\begin{aligned}\omega(\Psi(\xi_1,\eta_1)...\Psi(\xi_{2n},\eta_{2n})) &= \mathrm{Pf}((\omega(\Psi(\xi_i,\eta_i)\Psi(\xi_j,\eta_j)))_{ij}) \\ &= \sum_{\substack{J,K\in D_{I_{2n}},\\ J\sqcup K=I_{2n},\\ |J|=|K|,J<K}}(-1)^{\binom{n}{2}}\varepsilon(J,K)\prod_{i=1}^{n}\omega(\Psi(\xi_{j_i},\eta_{j_i})\Psi(\xi_{k_i},\eta_{k_i})).\end{aligned} \tag{10}$$

Here, $I_{2n} = \{1,...,2n\}$ and $D_{I_{2n}}$ denotes the ordered subsets of I_{2n}. In particular, the sum runs over disjoined partitions of I_{2n} into equally sized ordered subsets $J = \{j_1,...,j_n\}$, $K = \{k_1,...,k_n\}$ such that $j_i < k_i$, $i = 1,...,n$. The sign $\varepsilon(J,K)$ is given by the signature of the permutation $\binom{I}{JK} = \binom{1,...,2n}{j_1,...,j_n,k_1,...,k_n}$. For the infinite-length transverse-field Ising spin chain at criticality, the relevant two-point functions of the self-dual field are determined by Eq. (14) in the main document:

$$\begin{aligned}\omega(\Psi(0,i\delta_j)\Psi(\delta_{j'},0)) &= 2\Re\big(\omega(a_j a^\dagger_{j'}) - \omega(a^\dagger_j a^\dagger_{j'})\big) \\ &= \tfrac{1}{2\pi}\int dk\,|\hat{s}(k)|^2\big(\cos((j-j')k) + \mathrm{sign}(k)\sin((j-j')k)\big), \\ \omega(\Psi(0,i\delta_j)\Psi(0,i\delta_{j'})) &= -2i\Im\big(\omega(a_j a^\dagger_{j'}) - \omega(a^\dagger_j a^\dagger_{j'})\big) = 0, \\ \omega(\Psi(\delta_j,0)\Psi(\delta_{j'},0)) &= 2i\Im\big(\omega(a_j a^\dagger_{j'}) + \omega(a^\dagger_j a^\dagger_{j'})\big) = 0.\end{aligned} \tag{11}$$

As only the mixed correlations between $\Psi(0,i\delta_j)$ and $\Psi(\delta_{j'},0)$ are non-vanishing, the Pfaffian in (10) can be further reduced. Specifically, the two-point spin-spin correlation function can, thus, be evaluated in terms of a Toeplitz determinant [7, 8]:

$$\omega(\sigma^{(3)}_j\sigma^{(3)}_{j'}) = \det\begin{pmatrix} C^{(3)}_{-1} & C^{(3)}_{-2} & ... & C^{(3)}_{j-j'} \\ C^{(3)}_{0} & C^{(3)}_{-1} & ... & C^{(3)}_{j-j'+1} \\ ... & C^{(3)}_{0} & ... & ... \\ C^{(3)}_{j'-j-2} & ... & ... & C^{(3)}_{-1}\end{pmatrix}, \tag{12}$$

where $C^{(3)}_{j-j'} = \omega(\Psi(0, i\delta_j)\Psi(\delta_{j'}, 0)) = -\omega(\Psi(\delta_j, 0)\Psi(0, i\delta_{j'})) = C^{(3)}_{j'-j}$.

The evaluation of dynamical spin-spin correlation functions corresponding to real-time analytic continuations of the spin-spin correlators of the two-dimensional Ising model in infinite-volume requires additional effort (cp. Eq. (4) in the main document). To this end, following [2] we introduce *tail* and *string operators*,

$$T_M = \prod_{-M\leq l\leq 0} \sigma^{(1)}_l = \prod_{-M\leq l\leq 0} [a^\dagger_l, a_l] = \prod_{-M\leq l\leq 0} \Psi(\delta_l, 0)\Psi(0, i\delta_l), \tag{13}$$

$$S_j = \begin{cases} \prod_{0<l<j} [a^\dagger_l, a_l] \ : \ j > 0 \\ \prod_{j\leq l\leq 0} [a^\dagger_l, a_l] \ : \ j \leq 0 \end{cases},$$

which entails,

$$\sigma^{(3)}_j = T_M S_j (a_j + a^\dagger_j), \tag{14}$$

to understand the relation between $\mathcal{P}_M$ and $\mathfrak{A}_M$ via the Jordan-Wigner transformation (1) in the infinite-length limit $M \to \infty$ (see also [9, Ex. 6.2.14]). Although, T_M does not exist in this limit, the induced order-2 quasi-free automorphism by its adjoint action $\Theta_{M|-}(a_j) = \mathrm{Ad}_{T_M}(a_j) = \mathrm{sign}(j)a_j$, where $\mathrm{sign}(j) = \begin{cases} +1 \ : \ 0 < j \leq M-1 \\ -1 \ : \ -M \leq j \leq 0 \end{cases}$ remains well-defined (denoted by Θ_-). Therefore, $\mathcal{P}$ and $\mathfrak{A}$ can be realized as subalgebras of the crossed product $\hat{\mathfrak{A}} = \mathfrak{A} \rtimes_{\theta_-} \mathbb{Z}_2$, i.e., the algebra generated by $\mathfrak{A}$ and a self-adjoint unitary T such that:

$$\Theta_-(a(\xi)) = T a(\xi) T, \qquad \xi \in \mathfrak{h}. \tag{15}$$

Explicitly, the Pauli algebra $\mathcal{P}$ is given by (2) with T_M replaced by T, i.e., $\mathcal{P} = \mathfrak{A}^{(0)} + T\mathfrak{A}^{(1)}$, where $\mathfrak{A}^{(1)}$ is the odd subalgebra with respect to the $\mathbb{Z}_2$-grading Θ (see (7)). The infinite-length dynamics τ_t of the Hamiltonian H given in Eq. (10) in the main document exists on $\mathcal{P}$ by general methods for spin systems [9, Ex. 6.2.14] as well as on $\mathfrak{A}$, where it is quasi-free for the self-dual fields, $\tau_t(\Psi(\xi, \eta)) = \Psi(e^{ith}(\xi, \eta))$, and given by the diagonal one-particle Hamiltonian,

$$h(\theta) = 2\begin{pmatrix} 0 & -i\overline{z_\theta} \\ iz_\theta & 0 \end{pmatrix}, \qquad \theta \in [-\pi, \pi), \tag{16}$$

in momentum space. Thus, to evaluate the dynamical spin-spin correlation functions we essential need to understand the dynamics of the tail operator T. Because of the identity,

$$\sigma^{(3)}_j \tau_t(\sigma^{(3)}_{j'}) = (a_j + a^\dagger_j) S_j T \tau_t(T) \tau_t(S_{j'}(a_{j'} + a^\dagger_{j'})), \tag{17}$$

we essentially need to control the expression $T\tau_t(T)$ which is given by perturbation theory for inner automorphisms [10, Thm. 3.1.33]:

$$
\begin{aligned}
T\tau_t(T) &= \lim_{M\to\infty} Te^{itH_M}Te^{-itH_M} = \lim_{M\to\infty} e^{it\Theta_-(H_M)}e^{-itH_M} \\
&= \sum_{n=0}^{\infty} i^n \int_0^t dt_1 \int_0^{t_1} dt_2 \ldots \int_0^{t_{n-1}} dt_n \, \tau_{t_n}(P) \ldots \tau_{t_1}(P),
\end{aligned}
\tag{18}
$$

where $P = \lim_{M\to\infty} \Theta_-(H_M) - H_M = 2t^{(3)}(a_0 - a_0^\dagger)(a_1 + a_1^\dagger)$. With a similar strategy, it is possible to control expressions of the form,

$$
\alpha^m(T)\tau_t(\alpha^m(T)) = \lim_{M\to\infty} \lim_{N\to\infty} e^{it\alpha^m(T_N)H_M\alpha^m(T_N)}e^{-itH_M}, \tag{19}
$$

corresponding to perturbations $P^{(m)} = \lim_{M\to\infty} \lim_{N\to\infty} \alpha^m(T_N)H_M\alpha^m(T_N) - H_M$, appearing in the evaluation of dynamical spin-spin correlation functions.

ERROR BOUNDS: FERMIONIC CORRELATION FUNCTIONS

The explicit upper bound on the error δ in Eq. (17) in the main document appearing in the approximation of fermionic correlation functions, Eq. (16) in the main document, can be efficiently derived using the self-dual fields $\Psi(\xi, \eta) = a(\xi - i\eta) + a^\dagger(\overline{\xi + i\eta})$. It follows from the combinatorial formula for the evaluation of quasi-free states in terms of Pfaffians (10) that it is sufficient to derive the bound for two-point correlations functions which can be evaluated in terms of the one-particle space inner product of $\mathfrak{h} \oplus \mathfrak{h}$ and the one-particle covariance operator C_β determined by Eq. (12) in the main document:

$$
C_\beta = \begin{pmatrix} \mathbb{1} & i(\mathbb{1} - 2(C_\beta^{(1)} + C_\beta^{(2)})) \\ -i(\mathbb{1} - 2(C_\beta^{(1)} + C_\beta^{(2)})) & \mathbb{1} \end{pmatrix} = 2(e^{\beta h} + \mathbb{1})^{-1}, \tag{20}
$$

where h is the one-particle Hamiltonian (16). At criticality, i.e, $t^{(1)} = t^{(3)} = t$ and $\beta \to \infty$, the momentum-space kernel of the covariance reads,

$$
C_\infty(\theta, \theta') = 2\pi\delta(\theta - \theta') \underbrace{\begin{pmatrix} 1 & -i\frac{\overline{z_\theta}}{|z_\theta|} \\ i\frac{z_\theta}{|z_\theta|} & 1 \end{pmatrix}}_{= C_\infty(\theta)} = 2\pi\delta(\theta - \theta') \begin{pmatrix} 1 & -i\frac{1 - e^{-i\theta}}{2|\sin(\frac{1}{2}\theta)|} \\ i\frac{1 - e^{i\theta}}{2|\sin(\frac{1}{2}\theta)|} & 1 \end{pmatrix}. \tag{21}
$$

which becomes,

$$C(k,k') = 2\pi\delta(k-k') \underbrace{\begin{pmatrix} 1 & \mathrm{sign}(k) \\ \mathrm{sign}(k) & 1 \end{pmatrix}}_{=C(k)}, \tag{22}$$

in the scaling limit at criticality according to Eq. (14) in the main document. Putting everything together, the approximation of two-point correlation functions of Ψ reads as follows:

$$\begin{aligned} &|\omega^{(m)}(\Psi_{t_1^{(0)}}(\xi_1,\eta_1)\Psi_{t_2^{(0)}}(\xi_2,\eta_2)) - \omega(\Psi_{t_1}(\xi_1,\eta_1)\Psi_{t_2}(\xi_2,\eta_2))| \\ &= |\langle R^m(\xi_1,\eta_1), C_\infty e^{i(t_2^{(0)}-t_1^{(0)})h} R^m(\bar{\xi}_2,\bar{\eta}_2)\rangle - \langle R^{(\infty)}(\xi_1,\eta_1), C e^{i(t_2-t_1)h^{(\infty)}} R^{(\infty)}(\bar{\xi}_2,\bar{\eta}_2)\rangle|, \end{aligned} \tag{23}$$

where $R^{(\infty)}\xi = \xi * s$ is the asymptotic one-particle isometry as defined below Eq. (15) in the main document, and $h^{(\infty)}$ is the massless one-particle free-fermion Hamiltonian with momentum-space kernel $h^{(\infty)}(k) = 2k\sigma^{(1)}$. As explained in the main document, we expect the dynamical correlation functions of the m-times renormalized lattice model to approximated those of the scaling limit and the associated massless free-fermion dynamics only for effective lattice times of the order of the renormalization scale, i.e., $t_i^{(0)} = 2^m t_i$. Using the Cauchy-Schwarz inequality for Sobolev-type norms (with parameters $\gamma,\gamma_1,\gamma_2 > 0$), $\|C_\infty\| \leq 2$ (by (20)) and the unitarity of the dynamics, we find (using the condensed notation $\zeta_j = (\xi_j,\eta_j) \in \mathfrak{h}\otimes\mathbb{C}^2$, $j=1,2$):

$$\begin{aligned} &|\omega^{(m)}(\Psi_{t_1^{(0)}}(\zeta_1)\Psi_{t_2^{(0)}}(\zeta_2)) - \omega(\Psi_{t_1}(\zeta_1)\Psi_{t_2}(\zeta_2))| \\ &= |\langle R^{(\infty)}(\zeta_1), \Big(S_{2^{-m}} C_\infty e^{i(t_2^{(0)}-t_1^{(0)})h} S_{2^m} - C e^{i(t_2-t_1)h^{(\infty)}}\Big) R^{(\infty)}(\bar{\zeta}_2)\rangle| \\ &= |\tfrac{1}{2\pi}\int_{-\infty}^{\infty} dk\, |\hat{s}(k)|^2 \hat{\zeta}^\dagger_{1|k} \Big(C_\infty(2^{-m}k) e^{i(t_2^{(0)}-t_1^{(0)})h(2^{-m}k)} - C(k) e^{i(t_2-t_1)h^{(\infty)}(k)}\Big)\hat{\bar{\zeta}}_{2|k}| \\ &\leq |\tfrac{1}{2\pi}\int_{-\infty}^{\infty} dk\, |\hat{s}(k)|^2 \hat{\zeta}^\dagger_{1|k} \Big(C_\infty(2^{-m}k) - C(k)\Big) e^{i(t_2^{(0)}-t_1^{(0)})h(2^{-m}k)} \hat{\bar{\zeta}}_{2|k}| \\ &\quad + |\tfrac{1}{2\pi}\int_{-\infty}^{\infty} dk\, |\hat{s}(k)|^2 \hat{\zeta}^\dagger_{1|k} C(k) \Big(e^{i(t_2^{(0)}-t_1^{(0)})h(2^{-m}k)} - e^{i(t_2-t_1)h^{(\infty)}(k)}\Big)\hat{\bar{\zeta}}_{2|k}| \\ &\leq \tfrac{1}{2\pi}\Bigg[\int_{-\infty}^{\infty} dk\, \tfrac{|\hat{s}(k)|^{2(1-\gamma)}}{(1+|k|^2)^{\gamma_2}} \Big\|\Big(C_\infty(2^{-m}k) - C(k)\Big)\hat{\zeta}_{1|k}\Big\|^2 \int_{-\infty}^{\infty} dl\, (1+|l|^2)^{\gamma_2} |\hat{s}(l)|^{2\gamma} \Big\| e^{i(t_2^{(0)}-t_1^{(0)})h(2^{-m}l)} \hat{\zeta}_{2|l}\Big\|^2\Bigg]^{\frac{1}{2}} \\ &+ \tfrac{1}{2\pi}\Bigg[\int_{-\infty}^{\infty} dk\, (1+|k|^2)^{\gamma_1} |\hat{s}(k)|^{2(1-\gamma)} \Big\|C(k)\hat{\zeta}_{1|k}\Big\|^2 \int_{-\infty}^{\infty} dl\, \tfrac{|\hat{s}(l)|^{2\gamma}}{(1+|l|^2)^{\gamma_1}} \Big\|\Big(e^{i(t_2^{(0)}-t_1^{(0)})h(2^{-m}l)} - e^{i(t_2-t_1)h^{(\infty)}(l)}\Big)\hat{\zeta}_{2|l}\Big\|^2\Bigg]^{\frac{1}{2}} \end{aligned} \tag{24}$$

$$\leq \frac{1}{2\pi}\Bigg[\int_{-\infty}^{\infty} dk\ \tfrac{|\hat{s}(k)|^{2(1-\gamma)}}{(1+|k|^2)^{\gamma_2}} \Big\|\Big(C_\infty(2^{-m}k) - C(k)\Big)\hat{\zeta}_{1|k}\Big\|^2 \int_{-\infty}^{\infty} dl\ (1+|l|^2)^{\gamma_2}|\hat{s}(l)|^{2\gamma}\Big\|\hat{\zeta}_{2|l}\Big\|^2\Bigg]^{\frac{1}{2}}$$

$$+\frac{2^{\frac{1}{2}}}{2\pi}\Bigg[\int_{-\infty}^{\infty} dk\ (1+|k|^2)^{\gamma_1}|\hat{s}(k)|^{2(1-\gamma)}\Big\|\hat{\zeta}_{1|k}\Big\|^2 \int_{-\infty}^{\infty} dl\ \tfrac{|\hat{s}(l)|^{2\gamma}}{(1+|l|^2)^{\gamma_1}} \Big\|\Big(e^{i(t_2^{(0)}-t_1^{(0)})h(2^{-m}l)} - e^{i(t_2-t_1)h^{(\infty)}(l)}\Big)\hat{\zeta}_{2|l}\Big\|^2\Bigg]^{\frac{1}{2}}$$

$$= \frac{1}{2\pi}\Big\|\hat{s}^{2\gamma}\hat{\zeta}_2\Big\|_{H^{\gamma_2}(\mathbb{R})}\Bigg[\int_{-\infty}^{\infty} dk\ \tfrac{|\hat{s}(k)|^{2(1-\gamma)}}{(1+|k|^2)^{\gamma_2}} \Big\|\Big(C_\infty(2^{-m}k) - C(k)\Big)\hat{\zeta}_{1|k}\Big\|^2\Bigg]^{\frac{1}{2}}$$

$$+\frac{2^{\frac{1}{2}}}{2\pi}\Big\|\hat{s}^{2(1-\gamma)}\hat{\zeta}_1\Big\|_{H^{\gamma_1}(\mathbb{R})}\Bigg[\int_{-\infty}^{\infty} dk\ \tfrac{|\hat{s}(k)|^{2\gamma}}{(1+|k|^2)^{\gamma_1}} \Big\|\Big(e^{i(t_2^{(0)}-t_1^{(0)})h(2^{-m}k)} - e^{i(t_2-t_1)h^{(\infty)}(k)}\Big)\hat{\zeta}_{2|k}\Big\|^2\Bigg]^{\frac{1}{2}}$$

with $s_j(x) = s(x-j)$ and e_n, $n=1,2$ the standard basis vectors of $\mathbb{R}^2$, and where we used the fact the intermediate scaling map, $(R_m^{(\infty)}\hat{f})(k) = 2^{-\frac{m}{2}}\hat{s}(2^{-m}k)\hat{f}(k)$, is an isometry between the Hilbert spaces $L^2([-2^m\pi, 2^m\pi), (2^{m+1}\pi)^{-1})$ and $L^2(\mathbb{R}, (2\pi)^{-1})$, and defined the scaling transformation $(S_\lambda\hat{f})(k) = \hat{f}(\lambda k)$ in momentum space. Next, we evaluate the k-dependent norms inside the integrals in the last two lines of (24):

$$\Big\|\Big(C_\infty(2^{-m}k) - C(k)\Big)\hat{\zeta}_{1|k}\Big\|^2 = \Big|\tfrac{i(1-e^{i2^{-m}k})}{2\sin(\frac{1}{2}2^{-m}k)} - 1\Big|^2\Big\|\hat{\zeta}_{1|k}\Big\|^2, \tag{25}$$

$$\begin{aligned}\Big\|\Big(e^{i(t_2^{(0)}-t_1^{(0)})h(2^{-m}k)} - e^{i(t_2-t_1)h^{(\infty)}(k)}\Big)\hat{\zeta}_{2|k}\Big\|^2 \leq \Big(&|\cos(2t_0 t 2^{m+1}|\sin(2^{-(m+1)}k)|) - \cos(2t_0 t|k|)| \\ &+ |\sin(2t_0 t 2^{m+1}|\sin(2^{-(m+1)}k)|) - \sin(2t_0 t|k|)| \\ &+ |\tfrac{i(1-e^{i2^{-m}k})}{2\sin(\frac{1}{2}2^{-m}k)} - 1| + |1-\cos(2^{-m}k)|\Big)^2\Big\|\hat{\zeta}_{2|k}\Big\|^2,\end{aligned}$$

where $t_0 = t_2 - t_1$. Finally, we observe that,

$$\begin{aligned}\sup_{k\in\mathbb{R}} |k|^{-1}|\tfrac{i(1-e^{ik})}{2\sin(\frac{1}{2}k)} - 1| &= \tfrac{1}{2}, \\ \sup_{k\in\mathbb{R}} |k|^{-2}|1-\cos(k)| &= \tfrac{1}{2}, \\ \sup_{k\in\mathbb{R}} |k|^{-4}|\cos(2t_0 t 2^m 2|\sin(\tfrac{1}{2}k)|) - \cos(2t_0 t 2^m|k|)| &= 2^{2m}\tfrac{8}{3}(t_0 t)^2, \\ \sup_{k\in\mathbb{R}} |k|^{-3}|\sin(2t_0 t 2^m 2|\sin(\tfrac{1}{2}k)|) - \sin(2t_0 t 2^m|k|)| &= 2^m\tfrac{4}{3}t_0 t,\end{aligned} \tag{26}$$

which, together with (24) & (25), combine to the final estimate:

$$\begin{aligned}&|\omega^{(m)}(\Psi_{t_1^{(0)}}(\zeta_1)\Psi_{t_2^{(0)}}(\zeta_2)) - \omega(\Psi_{t_1}(\zeta_1)\Psi_{t_2}(\zeta_2))| \\ &\leq 2^{-m}\tfrac{2^{\frac{1}{2}}}{2\pi}\Bigg(\tfrac{2^{\frac{1}{2}}+1}{2^{\frac{1}{2}}}\sup_{k\in\mathbb{R}}|k|^{-1}|\tfrac{i(1-e^{ik})}{2\sin(\frac{1}{2}k)} - 1|\Big\|\hat{s}^{1-\gamma}\hat{\zeta}_1\Big\|_{H^1(\mathbb{R})}\Big\|\hat{s}^{\gamma}\hat{\zeta}_2\Big\|_{H^1(\mathbb{R})}\end{aligned} \tag{27}$$

$$
\begin{aligned}
&+2^{-m}\sup_{k\in\mathbb{R}}|k|^{-2}|1-\cos(k)|\left\|\hat{s}^{1-\gamma}\hat{\zeta}_1\right\|_{H^2(\mathbb{R})}\left\|\hat{s}^{\gamma}\hat{\zeta}_2\right\|_{H^2(\mathbb{R})}\\
&+2^{-2m}\sup_{k\in\mathbb{R}}|k|^{-3}|\sin(2t_0t2^m2|\sin(\tfrac{1}{2}k)|)-\sin(2t_0t2^m|k|)|\left\|\hat{s}^{1-\gamma}\hat{\zeta}_1\right\|_{H^3(\mathbb{R})}\left\|\hat{s}^{\gamma}\hat{\zeta}_2\right\|_{H^3(\mathbb{R})}\\
&+2^{-3m}\sup_{k\in\mathbb{R}}|k|^{-4}|\cos(2t_0t2^m2|\sin(\tfrac{1}{2}k)|)-\cos(2t_0t2^m|k|)|\left\|\hat{s}^{1-\gamma}\hat{\zeta}_1\right\|_{H^4(\mathbb{R})}\left\|\hat{s}^{\gamma}\hat{\zeta}_2\right\|_{H^4(\mathbb{R})}\Bigg)\\
\leq&2^{-m}\frac{2^{\frac{1}{2}}}{2\pi}\Bigg(\frac{2^{\frac{1}{2}}+1}{2^{\frac{1}{2}}2}\left\|\hat{s}^{1-\gamma}\hat{\zeta}_1\right\|_{H^1(\mathbb{R})}\left\|\hat{s}^{\gamma}\hat{\zeta}_2\right\|_{H^1(\mathbb{R})}+2^{-m}\tfrac{1}{2}\left\|\hat{s}^{1-\gamma}\hat{\zeta}_1\right\|_{H^2(\mathbb{R})}\left\|\hat{s}^{\gamma}\hat{\zeta}_2\right\|_{H^2(\mathbb{R})}\\
&+2^{-m}\tfrac{4}{3}t_0t\left\|\hat{s}^{1-\gamma}\hat{\zeta}_1\right\|_{H^3(\mathbb{R})}\left\|\hat{s}^{\gamma}\hat{\zeta}_2\right\|_{H^3(\mathbb{R})}+2^{-m}\tfrac{8}{3}(t_0t)^2\left\|\hat{s}^{1-\gamma}\hat{\zeta}_1\right\|_{H^4(\mathbb{R})}\left\|\hat{s}^{\gamma}\hat{\zeta}_2\right\|_{H^4(\mathbb{R})}\Bigg)\\
\leq&2^{-m}C_T\max_{\gamma_1+\gamma_2\in\{1,2,3,4\}}\left\|\hat{s}^{1-\gamma}\hat{\zeta}_1\right\|_{H^{\gamma_1}(\mathbb{R})}\left\|\hat{s}^{\gamma}\hat{\zeta}_2\right\|_{H^{\gamma_2}(\mathbb{R})},
\end{aligned}
$$

for $|t_0|\in[0,T]$, a constant $C_T>0$ and some free parameter $\gamma>0$. We note that the Sobolev-type norm are finite for sufficiently regular scaling functions $s\in C^r(\mathbb{R})$. Moreover, inspecting the penultimate line of (27) closely, we find that we can even choose the maximal continuum time to scale as $T\lesssim 2^mT_0$ for some $T_0>0$, i.e., the approximation error $\delta=\delta(m,T)$ vanishes in the limit $m\to\infty$ as long as $2^{-m}T\to 0$. In particular, the effective lattice times $t_i^{(0)}$ only need to be asymptotically large compared to the continuum times t_i, at the cost of an approximation error δ vanishing at least with the inverse asymptotics.

---

[1] T. J. Osborne and A. Stottmeister, Operator algebraic renormalization and tensor network renormalization, in preparation (2023).

[2] H. Araki, On the XY-model on two-sided infinite chain, Publications of the Research Institute for Mathematical Sciences **20**, 277 (1984).

[3] I. Daubechies, *Ten Lectures on Wavelets*, CBMS-NSF Regional Conference Series in Applied Mathematics, Vol. 61 (SIAM, 1992).

[4] G. Evenbly and S. R. White, Representation and design of wavelets using unitary circuits, Physical Review A: Atomic, Molecular, and Optical Physics **97**, 052314 (2018).

[5] T. D. Schultz, D. C. Mattis, and E. H. Lieb, Two-dimensional Ising model as a soluble problem of many fermions, Reviews of Modern Physics **36**, 856 (1964).

[6] D. E. Evans and Y. Kawahigashi, *Quantum symmetries on operator algebras*, Oxford Mathematical Monographs (The Clarendon Press, Oxford University Press, New York, 1998) pp.

xvi+829, oxford Science Publications.

[7] E. W. Montroll, R. B. Potts, and J. C. Ward, Correlations and Spontaneous Magnetization of the Two-Dimensional Ising Model, Journal of Mathematical Physics **4**, 308 (1963).

[8] Suzuki, Sei and Inoue, Jun-ichi and Chakrabarti, Bikas K., *Quantum Ising phases and transitions in transverse Ising models*, 2nd ed., Lecture Notes in Physics, Vol. 862 (Springer, 2012).

[9] O. Bratteli and D. W. Robinson, *Operator Algebras and Quantum Statistical Mechanics 2: Equilibrium States, Models in Quantum Statistical Mechanics*, 2nd ed., Theoretical and Mathematical Physics (Springer Berlin, Heidelberg, 1997).

[10] O. Bratteli and D. W. Robinson, *Operator Algebras and Quantum Statistical Mechanics 1: C^*-and W^*-Algebras, Symmetry Groups, Decomposition of States*, 2nd ed., Theoretical and Mathematical Physics (Springer Berlin, Heidelberg, 1987).
